# Simultaneous quarter-wave plate and half-mirror operation through a highly flexible single layer anisotropic metasurface

**DOI:** 10.1038/s41598-017-15279-8

**Published:** 2017-11-22

**Authors:** M. Ismail Khan, Farooq A. Tahir

**Affiliations:** Research Institute for Microwave and Millimeter-wave Studies, National University of Sciences and Technology (NUST), Islamabad, Pakistan

## Abstract

A highly flexible single-layer metasurface manifesting quarter-wave plate as well as half-mirror (1:1 beam-splitter) operation in the microwave frequency regime is being presented in this research. The designed metasurface reflects half power of the impinging linearly polarized electromagnetic wave as circularly polarized wave while the remaining half power is transmitted as circularly polarized wave at resonance frequency. Similarly, a circularly polarized incident wave is reflected and transmitted as linearly polarized wave with equal half powers. Moreover, the response of the metasurface is quite stable against the variations in the incidence angle up to 45°. The measurements performed on the fabricated prototype exhibit a good agreement with the simulation results. The compact size, flexible structure, angular stability and two in one operation (operating as a quarter-wave plate and beam-splitter at the same time) are the main characteristics of the subject metasurface that makes it a potential candidate for numerous applications in communication and miniaturized and conformal polarization control devices.

## Introduction

The control and manipulation of polarization state of electromagnetic (EM) waves is of central interest in the scientific community as it plays a key role in a wide range of applications such as optical sensing, contrast imaging and optical and microwave communications. The conventional techniques such as optical activity of crystals, the Brewster effect and the Faraday effect can be applied for polarization control; however, the devices based on such conventional techniques are largely handicapped due to their bulky size, narrow bandwidth and incident angle dependent response. To overcome these limitations, researchers are engaged in designing artificial structures whose electromagnetic properties can be tailored through a proper design of their shape, selection of their material and geometrical arrangement of their constituent elements. In this regard, the 2D metasurfaces^1^ have been extensively investigated for polarization control due to their obvious advantages over 3D metamaterials such as compact size, ease of fabrication and cost effectiveness.

In the last few years, metasurfaces have been successfully applied for manipulating the polarization of the electromagnetic waves in microwave^2–4^, terahertz^5–7^ and visible^8–10^ frequency regimes both in transmission^11–14^ and reflection^15–17^ modes. The polarization conversion capabilities of the metasurface originate from the anisotropy^18–22^ and intrinsic^23–25^ or extrinsic^26,27^ chirality of the unit cell. Efficient quarter-wave plate designs have been reported for infra-red^28^ and visible^29^ frequency regimes. Quarter-wave plate design using cut-wire-pair metamaterials are proposed^30^ for operation at terahertz frequencies. Wideband linear-to-circular polarization conversion is demonstrated using a bilayer metasurface for functioning in the microwave regime^31^.

Another important optical component is a beam splitter which plays a crucial role in numerous applications such as interferometry, Fourier-transform spectroscopy, optical switching and routing and isolation in optical communication systems. Conventional beam splitters are severely handicapped due to their large thickness, high power loss and high cost. A silicon based beam splitter is reported for infra-red and terahertz radiations^32^. An ultrathin broadband terahertz beam splitter is designed using low density polyethylene coated with silver^33^. A frequency selective surface (FSS) based beam splitter is reported for operation in microwave frequency regime^34^.

Although separate designs for polarization control and beam-splitting operations are available in the literature; however, metasurfaces with multiple functionalities in a single design are highly desirable. Such multifunctional metasurfaces not only miniaturize the size but also reduce the cost and complexity of the overall optical system by replacing multiple optical components through a single component. In this perspective, in the recent literature, a multifunctional metasurface exhibiting both half- and quarter-wave plate operation has been designed using bilayer anisotropic metasurface^35^. A plasmonic metasurface is theoretically and numerically investigated for both quarter-wave plate and half-mirror (1:1 beam splitter) operation in the visible frequency regime^36^. However, as the design is based on plasmonic material, therefore, it could not be rescaled for operation in the microwave regime. Another single layer metamaterial based design is theoretically and numerically presented to exhibit both quarter-wave plate and half-mirror operation in the microwave regime^37^. However, the proposed design is too complex to be fabricated.

In this article, the authors proposed, realized and experimentally demonstrated an ultrathin single layer metasurface achieving both quarter-wave plate and half-mirror operation in the microwave regime. The metasurface consists of an ultrathin flexible substrate on the top of which a two-dimensional periodic array of subwavelength metallic split-ring-resonators (SRRs), with a cross-element placed inside the SRRs, is printed. The proposed metasurface achieves linear-to-circular and circular-to-linear polarization transformation with half transmission and half reflection. Furthermore, the metasurface manifests a robust response to the variations in the incidence angle upto 45°. Owing to its twofold operation, compact size, angular stability and flexible structure, the designed metasurface is a promising candidate for telecommunication and miniaturized conformal polarization control devices.

## Results

### Geometrical Configuration

**Figure 1:**
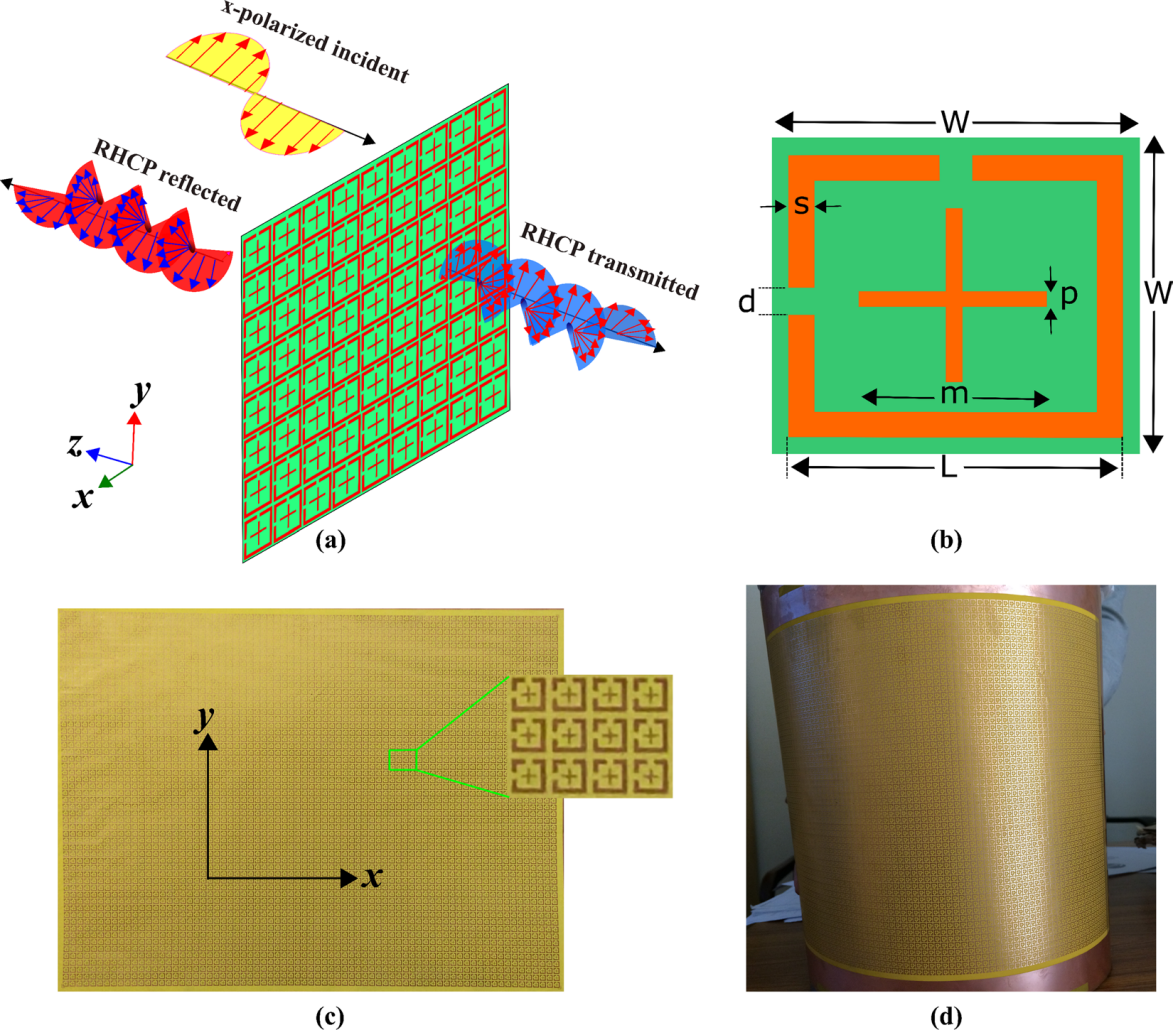
(**a**) A general schematic diagram of the proposed metasurface (**b**) Unit cell (**c**) Photograph of the fabricated metasurface. (**d**) A cylindrical object covered with fabricated sample to demonstrate flexibility.

A generalized schematic diagram of the polarization conversion metasurface is presented in Fig. 1(a).

It consists of two dimensional periodic array of the metallic unit cells printed on one side of the flexible polyimide substrate having thickness of 0.06 mm; nothing is printed on the other side of the substrate. The unit cell of the proposed metasurface is shown in Fig. 1(b). It consists of an SRR which has two equal-gap slits placed within its perpendicular sides; a cross-element is also printed inside the SRR. The unit cell is replicated along *x*- and *y*-axis with an equal periodicity of 3.5 mm. Figure 1(c) shows a photograph of the fabricated sample with an overall size of 12 × 12 inch. As the designed metasurface is fabricated on flexible substrate, therefore, it is conformal to any geometrical shape. To show the flexibility of the structure, a cylindrical object is covered with designed metasurface as shown in Fig. 1(d).

The reflected and transmitted fields generally consist of both *x*- and *y*-polarized components even if the incident field has only one component. The transmission coefficient *T*_*ij*_ is defined as the ratio of the transmitted field with polarization *i* to the incident field with polarization *j*. Similarly, the reflection coefficient *R*_*ij*_ is defined as the ratio of the reflected field with polarization *i* to the incident field with polarization *j*. The linear polarizations are labeled as *x* and *y* while the circular polarizations are denoted by “+” (right-handed circular polarization) and “–” (left-handed circular polarization). The incident and transmitted fields are related through Jones transmission matrix **T** in Cartesian basis:

1 $${\bf{T}}=(\begin{array}{cc}{T}_{xx} & {T}_{xy}\\ {T}_{yx} & {T}_{yy}\end{array})$$

The transmission matrix may also be written in circular basis:

2 $$\,{{\bf{T}}}_{{\bf{C}}{\bf{P}}}=(\begin{array}{cc}{T}_{++} & {T}_{+-}\\ {T}_{-+} & {T}_{--}\end{array})=\frac{1}{2}(\begin{array}{cc}{T}_{xx}+{T}_{yy}+i({T}_{xy}-{T}_{yx}) & {T}_{xx}-{T}_{yy}-i({T}_{xy}+{T}_{yx})\\ {T}_{xx}-{T}_{yy}+i({T}_{xy}+{T}_{yx}) & {T}_{xx}+{T}_{yy}-i({T}_{xy}-{T}_{yx})\end{array})$$

### Simulation Results

Metasurface shown in Fig. 1 is designed through full wave numerical simulations using Ansys HFSS. The optimized physical dimensions of the unit cell are, in millimeters: W = 3.5, L = 3, S = 0.5, d = 0.5, p = 0.25, m = 1.5 and thickness of the substrate is 0.06 mm. The dielectric used is polyimide with relative permittivity 3.4 and loss tangent of 0.004 while the material used for SRRs and ground plane is copper with conductivity 5.8 × 10^7^ S/m and thickness of 0.035 mm.

**Figure 2:**
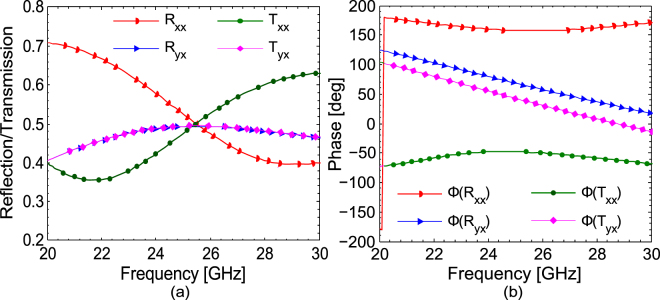
Co- and cross-polarized reflection and transmission coefficients for *x*-polarization (**a**) magnitude (**b**) phase.

The co- and cross-polarized reflection and transmission coefficients under normal incidence when the incident field is *x*-polarized are shown in Fig. 2(a). It can be seen from Fig. 2(a), that the co- (*T*_*xx*_) and cross-polarized (*T*_*yx*_) transmission coefficients attain the same value of 0.5 at the resonance frequency of 25 GHz. Moreover, the magnitude of co- (*R*_*xx*_) and cross-polarized (*R*_*yx*_) reflection coefficient is also 0.5 at 25 GHz. The phase of the reflection and transmission coefficients is shown in Fig. 2(b). The simulation results presented in Fig. 1 show that half power is reflected (as ${{R}_{xx}}^{2}+{{R}_{yx}}^{2}=0.5$) while the other half is transmitted (as ${{T}_{xx}}^{2}+{{T}_{yx}}^{2}=0.5$) at 25 GHz. The metasurface absorbs zero power, as $1-{{R}_{xx}}^{2}-{{R}_{yx}}^{2}-{{T}_{xx}}^{2}-{{T}_{yx}}^{2}=0,$ and hence acts as a lossless structure in the operating band.

The response of the metasurface for *y*-polarized incident wave can be predicted based on the geometrical symmetries in the unit cell. Although, the unit cell structure lacks fourfold rotational (C4) symmetry; however, it has mirror symmetry along *v*-axis or $x+y=0$ plane. The mirror operation along this plane can be represented by transformation matrix:

3 $${\bf{M}}=(\begin{array}{cc}0 & -1\\ -1 & 0\end{array})$$

**Figure 3:**
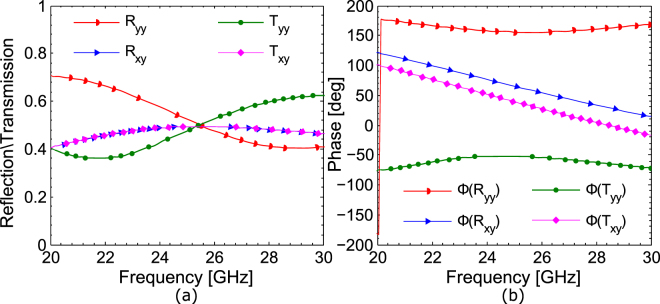
Co- and cross-polarized reflection and transmission coefficients for *y*-polarization (**a**) magnitude (**b**) phase.

The mirror symmetry ensures that ***MTM*** ^−1^ = ***T***, which after some algebraic manipulation, gives *T*_*xx*_ = *T*_*yy*_ and *T*_*yx*_ = *T*_*xy*_. This can also be verified from the simulation results, shown in Fig. 3, for *y*-polarized incident waves under normal incidence. Similar to the case of *x*-polarization, the co- and cross-polarized transmission and reflection coefficients reaches to a common value of 0.5 at 25 GHz. Furthermore, the magnitude and phase of the reflection and transmission coefficients for *y*-polarization are similar to the corresponding reflection and transmission coefficients for *x*-polarization.

**Figure 4:**
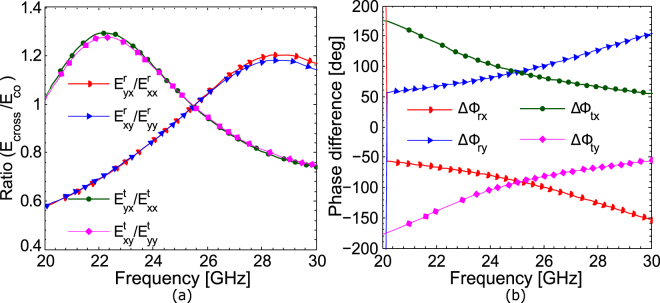
(**a**) Ratio of the cross- and co-polarized fields. (**b**) Phase difference for normal incidence.

A birefringent crystal manipulates the polarization state of the impinging wave by retarding one linearly polarized component with respect to the other resulting in some phase difference between them. If phase difference is 90° and ratio of orthogonal components of the field is 1, then the crystal exhibits quarter-wave plate operation converting a linearly polarized wave to a circularly polarized wave. The performance as a quarter-wave plate is acceptable^32^ if the ratio is within the range of 0.85–1.15 and the phase difference lies within 85°–95°. Here, we used the amplitude bandwidth as the frequency range within which the ratio of the fields lies within 0.86–1.14 and the phase bandwidth as the range of frequencies for which the absolute value of the phase difference is within 85°–95°. The intersection of the amplitude and phase bandwidth gives the operating bandwidth of the quarter-wave plate. The magnitude of the ratio of the cross- and co-polarized fields and their phase difference are shown in Fig. 4(a) and (b) respectively. It can be seen from Fig. 4, the amplitude bandwidth criterion is satisfied over frequency range 24–26.8 GHz while the phase bandwidth criterion is fulfilled for 24.6–25.6 GHz.

To investigate the response of the metasurface for circularly polarized incident waves, we insert *T*_*xx*_ = *T*_*yy*_ and *T*_*yx*_ = *T*_*xy*_ into Eq. (2), the transmission coefficients for circular polarization becomes ${T}_{++}={T}_{--}={T}_{xx}\,\,$and ${T}_{+-}=-{T}_{-+}=-i{T}_{yx}\,$. This means that, at resonance frequency, the co-polarized (*T*_++_and *T*_−−_) and cross-polarized (*T*_+−_ and *T*_−+_) circular transmission coefficients attain same magnitude equal to 0.5 implying that half power of a RHCP wave is transmitted both as RHCP and LHCP wave. As, a linearly polarized wave can be expressed as a superposition of RHCP and LHCP wave, therefore, an incident circularly polarized wave is reflected and transmitted as linearly polarized wave with half power in each.

### Angular Stability

**Figure 5:**
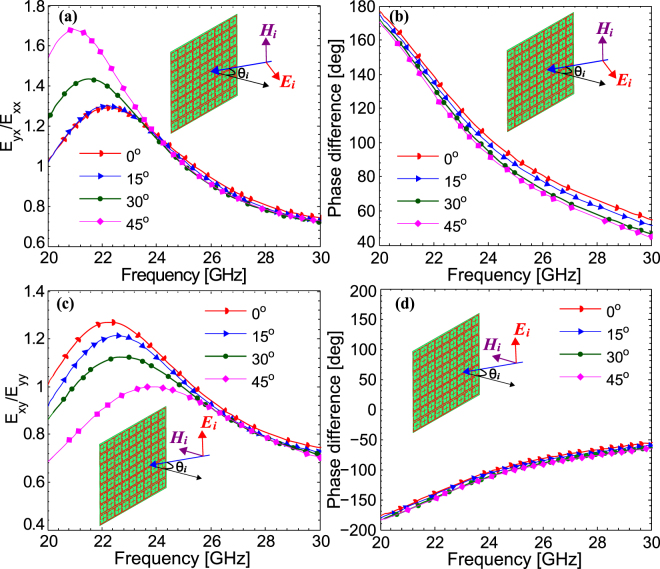
(**a**) Ratio of the cross- and co-polarized transmitted fields and (**b**) phase difference for TM polarization (*x*-polarization) (**c**) ratio of the cross- and co-polarized transmitted fields and (**b**) phase difference for TE polarization (*y*-polarization).

In order to be useful in a wide variety of applications, the response of the metasurface must be stable against variations in the incidence angle. The ratio of the transmitted fields and their phase difference for different incidence angles under *x*-polarization (transverse-magnetic (TM) polarization) is presented in Fig. 5(a) and (b) respectively. It can be seen from Fig. 5(a) and (b) that the response of the metasurface is quite robust to the variations in the incidence angle within the both amplitude and phase bandwidths. The small deviations in the response of the metasurface for large incidence angles under TM polarization are due to the electric field passing through the SRR loop which disturbs the current flow. The angular stability is not only demonstrated for *x*-polarization but also for *y*-polarization (TE-polarization) as shown in Fig. 5(c) and (d). For the case of TE polarization, small variations in the response of the metasurface result from the magnetic field which passes through the SRR loop at oblique incidence and disturbs the current flow. However, these variations are small enough and lie within acceptable range. The angular stability of the metasurface results from the small subwavelength unit cell size (0.29λ), small dielectric thickness (0.005 λ) where λ is the free space wavelength at 25 GHz and optimized design of the unit cell.

### Eigen-Polarizations

The Jones transmission coefficient matrix at the resonance frequency, 25 GHz, can be written as:

$${\bf{T}}=0.5(\begin{array}{cc}{e}^{-i\frac{\pi }{4}} & {e}^{i\frac{\pi }{4}}\\ {e}^{i\frac{\pi }{4}} & {e}^{-i\frac{\pi }{4}}\end{array})$$

**Figure 6:**
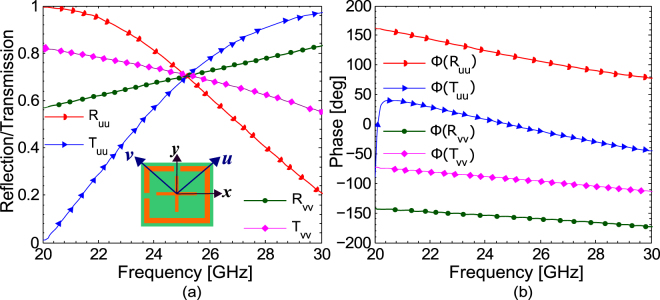
(**a**) Magnitude of the reflection and transmission coefficients and (**b**) phase difference for *u*- and *v*-polarization.

The linearly independent eigenvectors for matrix **T** are $${\boldsymbol{u}}={(\begin{array}{cc}1 & 1\end{array})}^{T}$$ and $${\boldsymbol{v}}={(\begin{array}{cc}-1 & 1\end{array})}^{T}$$ with eigenvalues $\sqrt{2}/2$ and $\sqrt{2}/2{e}^{-i\frac{\pi }{2}}$ respectively. Eigenvectors ***u*** and ***v*** are at ±45° to *x*- and *y*-axis as shown in the inset of Fig. 6(a). The eigenvalues indicate that that eigen-polarizations (*u*- and *v*-polarization) are transmitted with half power without any change of polarization state. Moreover, the phase of the *u*-polarization at the exit of the metasurface is 0° while it is −90° for *v*-polarization. To further verify this, simulations were carried out for *u-* and *v-*polarizations under normal incidence. Figure 6(a) and (b) show the magnitude and phase of the reflection and transmission coefficients respectively for eigen-polarizations. It can be seen from Fig. 6 that the magnitude of the co-polarized transmission coefficient both for *u*- and *v*-polarizations has same value equal to 0.707 while the phase achieves the value of 0° and −90° for both *u*- and *v*-polarizations respectively. Since, *x*- or *y*-polarized fields can be decomposed into *u*- and *v*-components, ${E}_{x}\hat{x}=\frac{\sqrt{2}}{2}{E}_{x}\hat{u}-\frac{\sqrt{2}}{2}{E}_{x}\hat{v}$ and ${E}_{y}\hat{y}=\frac{\sqrt{2}}{2}{E}_{y}\hat{u}+\frac{\sqrt{2}}{2}{E}_{y}\hat{v}$, therefore, after transmission through the metasurface, *u*- and *v*-components have equal magnitudes and 90° phase difference resulting into circular polarization. The same discussion also holds for reflection coefficients, where a linearly polarized incident wave is also reflected as circularly polarized wave with half power. Therefore, for eigen-polarizations the proposed metasurface acts only as a lossless 1:1 beam splitter reflecting and transmitting half power without affecting the polarization state of the impinging electromagnetic wave.

### Frequency Tunability

**Figure 7:**
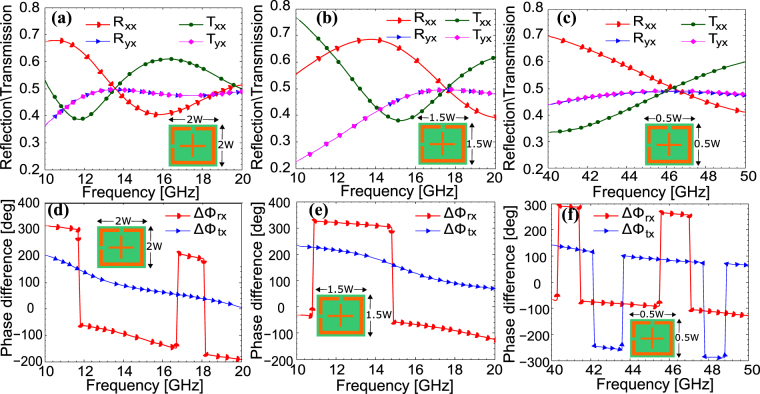
((**a**), (**b**) and (**c**)) Magnitude of the reflection and transmission coefficients when physical dimensions in *xy*-plane are scaled by (**a**) 2 (**b**) 1.5 and (**c**) 0.5. ((**d**), (**e**) and (**f**)) Phase difference when physical dimensions in *xy*-plane are scaled by (**d**) 2 (**e**) 1.5 and (**f**) 0.5.

An essential feature required for the reproducibility of any electromagnetic design for applications in various electromagnetic frequency regimes is its ability to control the operating frequency/frequencies through the physical dimensions of the structure. In order to investigate this, the proposed structure is simulated with different physical dimensions and the results are presented in Fig. 7. As it can be seen from Fig. 7(a) and (d), the operating frequency of the metasurface is lowered to 13.5 GHz when the physical dimensions of the unit cell in the *xy*-plane are scaled by two. Similarly, as shown in Fig. 7(b) and (e), when the physical dimensions in the *xy*-plane are scaled by 1.5, the operating frequency reaches to 17.5 GHz. Figure 7(c) and (f) show that the operating frequency is increased to 45.7 GHz as the physical dimensions in the *xy*-plane are scaled by 0.5. As a result of the above parameteric analysis, it can be concluded that the operating frequency band can be shifted to any desirable frequency by properly scaling the physical dimensions of the unit cell. The metasurface maintains both quarter-wave plate and half-mirror operation when the physical dimensions are scaled in the *xy*-plane and thus it can be easily scaled for terahertz, infra-red and visible frequency domain applications.

### Experimental Verification

**Figure 8:**
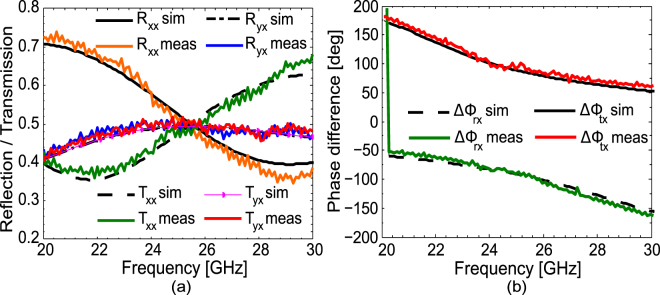
(**a**) Magnitude of the simulated and measured co- and cross-polarized reflection and transmission coefficients. (**b**) Simulation and measurement results for the phase difference.

In order to experimentally verify the proposed design, a prototype of the design was fabricated and tested in a fully anechoic chamber. The fabricated sample consists of 74 × 74 unit cells etched on the top of an ultrathin flexible polyimide substrate with a thickness of 0.006 mm. Two double-ridge broadband horn antennas (OBH100400) and a vector network analyzer (HP 8722 C) were used for the measurement of the reflection and transmission coefficients. For measuring the co-polarized coefficients, both transmitting and receiving antennas are placed along the same orientation (horizontal or vertical) while for cross-polarized measurements they are placed perpendicular to each other. The measured results for the magnitude of co- and cross-polarized reflection and transmission coefficients and phase difference between the orthogonal components are presented in Fig. 8(a) and (b) respectively. It can be seen from Fig. 8 that the measurement and simulation results are in good agreement with each other.

## Discussions

The authors have designed and experimentally demonstrated an ultrathin metasurface which acts not only as a quarter-wave plate both in transmission and reflection mode but also as a lossless 1:1 beam splitter. The designed metasurface reflects half power of the incident linearly polarized electromagnetic wave as a circularly polarized wave while the remaining half power is transmitted as a circularly polarized wave at resonance frequency. Similarly, a circularly polarized incident wave is reflected and transmitted as a linearly polarized wave with equal half power. Moreover, the metasurface manifests a robust response to the variations in the incidence angle due to its subwavelength unit cell size (0.29λ), small dielectric thickness (0.005 λ) where λ is the free space wavelength at 25 GHz and optimized unit cell dimensions. Furthermore, the designed metasurface can also act as a non-polarizing 1:1 beam splitter when the incident wave is polarized along ± 45° to the *x*- or *y*-axis. The proposed design combines the advantages of both quarter-wave plate and half mirror. Owing to its twofold operation, compact size, ease of fabrication, angular stability and flexible and lossless structure, the proposed metasurface is a promising candidate for numerous applications in telecommunication and miniaturized polarization control devices.

## Methods

The proposed design was simulated and optimized through numerical full wave simulations using Ansys HFSS. In the simulation setup, periodic boundary conditions (PBCs) are adopted in the transverse *xoy* plane while two Floquet ports are assigned on the top and bottom boundaries. Copper metal with a finite conductivity of 5.8 × 10^7^ S/m was used for the metallic part while the substrate part was assigned polyimide material with relative permittivity 3.4 and loss tangent 0.004. To practically realize and experimentally demonstrate the simulated design, a prototype of the proposed design was fabricated on 305 × 305 × 0.006 mm^3^ fully flexible polyimide sheet on the top of which 74 × 74 unit cells were etched using standard printed circuit board (PCB) techniques. Two double-ridge broadband horn antennas (OBH100400) and a vector network analyzer (HP 8722 C) were used for the measurement of the reflection and transmission coefficients. The whole experimental setup was placed in a fully anechoic chamber. First, co- and cross-polarized reflection coefficients were measured by placing the transmitting and receiving antennas on the same side and then transmission coefficients were measured by placing the transmitting antenna on one side while the receiving antenna on the other side of the metasurface. For measuring co-polarized coefficients both transmitting and receiving antennas are placed along the same orientation (horizontal or vertical) while for cross-polarized measurements they are placed perpendicular to each other.

### Data availability

The datasets generated during and/or analyzed during the current study are available from the corresponding author on reasonable request.
