# Author Correction: Simultaneous quarter-wave plate and half-mirror operation through a highly flexible single layer anisotropic metasurface

**DOI:** 10.1038/s41598-018-27269-5

**Published:** 2018-06-07

**Authors:** M. Ismail Khan, Farooq A. Tahir

**Affiliations:** Research Institute for Microwave and Millimeter-wave Studies, National University of Sciences and Technology (NUST), Islamabad, Pakistan

Correction to: *Scientific Reports* 10.1038/s41598-017-15279-8, published online 22 November 2017

The authors missed a previously published related article that reports a similar matrix design. This article is included below as Reference 1, and this reference and its discussion should be included in the Article as below.

In the Results section, under the subheading ‘Geometrical Configuration’,

“To show the flexibility of the structure, a cylindrical object is covered with designed metasurface as shown in Fig. 1(d).”

should read:

“To show the flexibility of the structure, a cylindrical object is covered with designed metasurface as shown in Fig. 1(d).

The authors feel the need to comment here on a design reported in [1] that is also based on split-ring resonator having two orthogonally located splits with in it. Alongside the apparent difference in overall unit cell geometries of the two designs, the design proposed by the authors differs in operating principle as well as in functions from [1]. The design presented in [1] is simple polarization rotator i.e., it exhibits half-wave plate operation; while our design manifests totally different behavior. Our design exhibits two different yet complex operations, i.e., quarter-wave plate as well as half-mirror (1:1 beam-splitter) operation. The half-mirror operation is itself very critical and is used in many practical applications of microwave and optical regime. Further, the design of [1] is bi-layer and fabricated on a rigid thick substrate (F4B) limiting its utility in low-profile as well as flexible polarization control devices. Our proposed design, in comparison, is single layer and made on an ultra-thin flexible substrate of polyimide. In brief, it is very challenging to achieve such a design presenting multiple functions on a single yet flexible dielectric layer, and this is accomplished by the design being presented in this paper.”
